# 虚拟支气管镜导航联合支气管超声导向鞘引导肺活检术在肺周围性病变诊断中的应用

**DOI:** 10.3779/j.issn.1009-3419.2019.03.01

**Published:** 2019-03-20

**Authors:** 士杰 李, 万璞 闫, 麦林 陈, 利 孙, 齐 吴, 克能 陈

**Affiliations:** 1 100142 北京，北京大学肿瘤医院暨北京市肿瘤防治研究所，恶性肿瘤发病机制及转化研究教育部重点实验室内镜中心 Key Laboratory of Carcinogenesis and Translational Research (Ministry of Education), Peking University Cancer Hospital Department of Endoscopy Center; 2 100142 北京，北京大学肿瘤医院暨北京市肿瘤防治研究所，恶性肿瘤发病机制及转化研究教育部重点实验室胸外一科 Key Laboratory of Carcinogenesis and Translational Research (Ministry of Education), Peking University Cancer Hospital The First Department of Thoracic Surgery; 3 100142 北京，北京大学肿瘤医院暨北京市肿瘤防治研究所，恶性肿瘤发病机制及转化研究教育部重点实验室医学影像科 Key Laboratory of Carcinogenesis and Translational Research (Ministry of Education), Peking University Cancer Hospital Department of Radiology; 4 100142 北京，北京大学肿瘤医院暨北京市肿瘤防治研究所，恶性肿瘤发病机制及转化研究教育部重点实验室病理科 Key Laboratory of Carcinogenesis and Translational Research (Ministry of Education), Peking University Cancer Hospital Department of Pathology

**Keywords:** 肺周围性病变, 支气管超声导向鞘, 虚拟支气管镜导航, Pulmonary peripheral lesions, Endobronchial ultrasonography with guide sheath, Virtual bronchoscopy

## Abstract

**背景与目的:**

虚拟支气管镜导航（virtual bronchoscopic navigation, VBN）联合支气管超声导向鞘技术（endobronchial ultrasonography with guide sheath, EBUS-GS）用于经支气管肺活检术（transbronchus lung biopsy, TBLB），能够减少甚至避免射线暴露。本研究拟评价VBN联合EBUS-GS对肺周围性病变（pulmonary peripheral lesions, PPLs）的诊断价值及安全性。

**方法:**

回顾性分析2016年1月-2017年12月在北京大学肿瘤医院内镜中心接受EBUS-GS-TBLB患者的临床病理资料，评价诊断率及安全性，并对影响诊断率的因素进行分析。

**结果:**

121例患者纳入本研究，男性65例，女性56例；平均年龄（58.8±10.3）岁。108例（89.3%）可在EBUS图像中显示，共有89例（73.5%）经EBUS-GS-TBLB获得明确诊断。EBUS-GS-TBLB对恶性病变的诊断率为82.5%。联合刷检、活检及肺泡灌洗中两种或三种方式取检的诊断率（87.2%）高于仅采用单一方式取检（58.8%）（*χ*^2^=6.084, *P*=0.014）。影响EBUS-GS-TBLB诊断率的因素包括超声探头位于病灶内部（*χ*^2^=20.372, *P*=0.000）、病灶位于肺野内带及中带（*χ*^2^=10.810, *P*=0.001）。1例（0.8%）患者术中出血量较多。

**结论:**

VBN联合EBUS-GS-TBLB是一种诊断PPLs安全且有效的方法。

## Abstract (en)

**Background and objective:**

Virtual bronchoscopic navigation (VBN) assisted endobronchial ultrasonography with guide sheath (EBUS-GS) has reduced the difficulty and even avoiding radiation exposure during performing transbronchus lung biopsy (TBLB). To evaluate the feasibility and safety of virtual bronchoscopic navigation assisted endobronchial ultrasonography with guide sheath for peripheral pulmonary lesions.

**Methods:**

We performed a retrospective analysis of the patients with PPLs who received VBN assisted EBUS-GS-TBLB in Peking University Cancer Hospital from January 2016 to December 2017. Their clinicopathologic data and complications were assessed.

**Results:**

A total of 121 patients were enrolled in the study. The patients included 65 men and 56 women, with a mean age of (58.8±10.3) years. A total of 121 PPLs were examined, and 108 lesions of which could be detected by EBUS. The overall diagnostic yield of EBUS-GS was 73.5%. The diagnostic yield of malignancy was 82.5%. The combination of transbronchial lung biopsy, brush smear and bronchoalveolar lavage fluid provided the greatest diagnostic yield (*χ*^2^=6.084, *P*=0.014). Factors that significantly affected and predicted diagnostic success were EBUS probe within the lesions (*χ*^2^=20.372, *P*=0.000) and PPLs located in the central two-thirds of the lung (*χ*^2^=10.810, *P*=0.001). 1 patient (0.8%) suffered from intraoperative bleeding which could be managed under endoscopy.

**Conclusion:**

VBN assisted EBUS-GS-TBLB for PPLs was an effective and safe procedure.

支气管肺癌（简称肺癌）是世界范围内发病率及死亡率最高的恶性肿瘤，其预后与临床分期密切相关^[1]^。2015年我国肺癌新发病例73.3万，死亡病例61.0万^[2]^，75%的肺癌患者诊断时已属晚期^[3]^，患者5年生存率仅为16.1%^[4]^。随着胸部计算机断层扫描（computed tomography, CT）筛查技术的普及，肺周围性病变（pulmonary peripheral lesions, PPLs）的检出率大幅提高，其中一部分为早期肺癌^[5]^。作为PPLs定性诊断的重要手段，CT引导下经胸壁针吸活检术（transthoracic needle aspiration, TTNA）和X线透视下经支气管肺活检术（transbronchus lung biopsy, TBLB）都增加了患者和医务人员辐射暴露风险。2004年Kurimoto^[6]^首次报道支气管超声导向鞘（endobronchial ultrasonography with guide sheath, EBUS-GS）技术联合TBLB用于PPLs的诊断。美国胸科医师协会（American College of Chest Physicians, ACCP）肺癌指南推荐EBUS技术用于PPLs的定性诊断^[7]^。近年来，虚拟支气管镜导航（virtual bronchoscopic navigation, VBN）技术降低了EBUS-GS操作难度，减少甚至避免了射线暴露，推动了EBUS-GS-TBLB的普及^[8]^。本研究回顾性分析EBUS-GS-TBLB联合VBN在无X线监视下用于PPLs诊断的有效性及安全性。

## 1 资料与方法

### 1.1 研究对象

回顾性分析2016年1月-2017年12月北京大学肿瘤医院内镜中心连续就诊的门诊及住院患者的临床病理资料。纳入标准：（1）胸部增强CT检查为PPLs；（2）既往支气管镜检查未见气管内病变，且行肺泡灌洗细胞学检查无明确诊断；（3）病例获得明确的病理诊断或临床诊断。排除标准：不能配合完成检查者。本研究获得北京大学肿瘤医院伦理委员会批准（批号：2016KT15），患者术前均签署知情同意书。

### 1.2 研究设备

260型内镜主机，BF-P260支气管镜（外径4.0 mm，操作孔道内镜2.0 mm），EU-ME1超声主机，MAJ-935小探头驱动器，UM-S20-17S环扫超声探头（20 MHz），引导鞘套装（K-201），以上设备均为日本Olympus公司产品。DirectPath^®^虚拟导航系统（Cybernet System Inc., Tokyo, Japan）。

### 1.3 研究方法

#### 1.3.1 操作方法

术前仔细阅读患者胸部高分辨率CT，将CT数据以DICOM格式导入DirectPath^®^系统，生成选择引导合适的引导路径。术前禁食8 h，禁水4 h，2%利多卡因10 mL雾化吸入局部麻醉。经一侧鼻孔置入气管镜，根据导航路径指示，到达目标支气管（或直至不能继续插入），经活检管道置入带鞘管的超声探头，根据超声图像，调整鞘管及探头位置，直至获得满意超声图像。固定鞘管并退出超声探头，经鞘管置入细胞刷、活检钳，进行刷检、活检（至少3次或获取满意组织块）及肺泡灌洗操作。若反复尝试无法获得病灶超声图像，则在支气管镜所到达的末端支气管开口进行肺泡灌洗操作。术后将收集的细胞学及组织学标本固定后送病理检查。术中鼻导管吸氧并监测患者心率、血压及末梢血氧饱和度，记录术中及术后出血、低氧血症等不良事件。

#### 1.3.2 病理结果判定

所有病理结果均由2位经验丰富的病理科医师判定。EBUS-GS-TBLB所获标本明确恶性诊断者定义为阳性；结果为良性疾病（如结核、结节病等）或无明确恶性诊断者定义为阴性。EBUS-GS-TBLB无明确诊断者建议接受CT引导下穿刺或胸腔镜手术等方式确诊，拒绝者每3个月复查胸部CT进行随诊。

### 1.4 统计学方法

采用SPSS 20.0统计软件进行分析。正态分布的计量资料以均数±标准差（Mean±SD）表示，采用*t*检验；计数资料以率表示，率的比较采用卡方检验，以*P* < 0.05为差异具有统计学意义。

## 2 结果

### 2.1 一般资料

121例患者共计121处病灶纳入本研究。患者一般资料见表 1。

**1:** 患者临床特征 Clinical characteristics of patients

Item	Data
Patient volume (*N*)	121
Gender (male:female)	65:56
Age (Mean±SD, yr)	58.8±10.3
Lesion location [*n*(%)]	
LUL	34 (28.1)
LLL	8 (6.7)
RUL	40 (33.1)
RML	14 (11.6)
RLL	25 (20.7)
Lesion size	
CT maximum dimeter (Mean±SD, mm)	25.4±11.8
CT maximum dimeter ≤30 mm [*n*(%)]	96 (79.3)
CT maximum dimeter ≤20 mm [*n*(%)]	50 (41.3)
LUL: Left upper lobe; LLL: Left lower lobe; RUL: right upper lobe; RML: right middle lobe; RLL: right lower lobe; CT: computed tomography.	

### 2.2 EBUS-GS检查情况

共有108例（89.3%）病灶可在EBUS图像中显示，其中68例探头位于病灶内部，40例探头位于病灶外侧边缘。其余13例（10.7%）病例中，10例无法探及病变，3例虽经反复调整鞘管位置，病变仍远离探头。所有病例均获得了满意的组织学或细胞学标本，其中组织学标本获取率为70.2%（85/121），1例因刷检后出血明显未行活检，其余35例经多次尝试无法获得组织学标本；细胞学标本获取率为97.5%（118/121），其余3例均因细胞学标本中混杂较多凝血块而未予送检。另有39例（32.2%）患者合并纵隔或肺门淋巴结肿大，同时接受了支气管内超声引导下经支气管针吸活检术（endobronchial ultrasound-guided transbronchial needle aspiration, EBUS-TBNA）或超声内镜引导下针吸活检术（endoscopic ultrasound-guided fine needle aspiration, EUS-FNA）。

### 2.3 诊断结果

共有89例通过EBUS-GS-TBLB确诊，包括鳞癌7例、腺癌46例、非小细胞癌11例（7例仅有细胞学标本，无法进一步分类；其余4例尽管获得组织学标本，但无法通过免疫组化染色进一步分类）、小细胞癌9例、恶性肿瘤无法分类9例（均为细胞学恶性诊断，其中3例同时获得的组织学标本为凝血样物或良性上皮组织）、结核3例、结肠癌2例、淋巴瘤1例、肉芽肿性炎1例；8例通过手术确诊，包括鳞癌1例、腺癌5例、肉瘤样癌1例、炎性改变1例；9例通过CT穿刺确诊，包括腺癌5例、炎性改变2例、结核1例、真菌感染1例；5例通过EBUS-TBNA明确诊断，包括腺癌4例，结肠癌1例；其余10例中，5例经抗炎治疗后病灶消失，4例随访过程中（随访时间21个月-32个月）病灶稳定，临床均考虑良性；1例85岁老年男性，经实验性口服盐酸埃克替尼1个月后复查CT，病变缩小约30%，临床考虑恶性。

对恶性病变而言，EBUS-GS-TBLB的诊断率为82.5%（85/103）。其中，51例组织学诊断恶性，78例细胞学诊断恶性。活检、刷检及灌洗三种取检手段的诊断率分别为68.0%、77.3%及62.1%。Within型病例刷检及灌洗诊断率（91.1%及73.1%）均高于Adjacent型（55.2%及45.5%），且差异具有统计学意义（*χ*^2^=12.872，*P*=0.000及*χ*^2^=7.264，*P*=0.007），但与Without型的差异不具有统计学意义（*P* > 0.05）。Within型病例活检诊断率高于Adjacent型，差异不具有统计学意义（*P* > 0.05）（表 2）。对于良性病变而言，EBUS-GS-TBLB的阴性预测值为50.0%（18/36），然而仅有4例（3例结核，1例肉芽肿性炎）通过EBUS-GS-TBLB获得了明确的良性病理诊断。

**2:** 恶性病灶中不同取检方式的诊断率分析 Analysis of diagnosis rate with different sampling methods in malignant lesions

	Within type (*N*=59)	Adjacent type (*N*=34)	Without type (*N*=10)	Total
Biopsy [% (*n*/*N*)]	74.1 (40/54)	52.4 (11/21)	-	68.0 (51/75)
Brushing [% (*n*/*N*)]	91.1 (41/45)	55.2 (16/29)	100.0 (1/1)	77.3 (58/75)
Lavage [% (*n*/*N*)]	73.1 (38/52)	45.5 (15/33)	60.0 (6/10)	62.1 (59/95)
Within type: the probe was located inside the lesion; Adjacent type: the probe was located on the lateral edge of the lesion; Without type: the probe was located far from or inaccessible for the lesion.				

### 2.4 影响EBUS-GS-TBLB对恶性病变诊断率的因素

93.2%的Within型及88.2%的Adjacent型病例接受了联合取检的模式，其比例高于Without型病变（10%），差异具有统计学意义（*χ*^2^=33.477，*P*=0.000及*χ*^2^=19.118，*P*=0.000）。联合活检、刷检及灌洗中两种或三种方式取检的诊断率为87.2%（75/86），高于仅采用单一方式取检的58.8%（10/17），差异具有统计学意义（*χ*^2^=6.084, *P*=0.014）（表 3）。此外，Within型病例诊断率为96.6%，高于Adjacent型及Without型，差异具有统计学意义（*χ*^2^=20.372, *P*=0.000）。CT中病灶位于肺野内带及中带者诊断率为90.4%，高于位于肺野外带者，差异具有统计学意义（*χ*^2^=10.810, *P*=0.001）。尽管病灶直径 > 20 mm、CT中可见支气管征的EBUS-GS-TBLB诊断率更高，但其差异不具有统计学意义（*P* > 0.05）。不同肺叶病灶诊断率间的差异不具有统计学意义（*χ*^2^=1.377, *P*=0.848）。在最终确诊的103例恶性病例中，前20例的EBUS-GS-TBLB诊断率（80.0%）略低，但差异不具有统计学意义（*χ*^2^=0.000, *P*=0.997）（表 4）。

**3:** 恶性病变采用联合取检手段对于诊断率影响的分析 Analysis of diagnosis rate with combined use of different sampling methods

	Single sampling methods [% (*n*/*N*)]				Combined sampling methods [% (*n*/*N*)]				Total [% (*n*/*N*)]
	Biopsy	Brushing	Lavage		Biopsy + Brushing	Biopsy + Lavage	Brushing + Lavage	All three methods	
Within type (*N*=59)	100.0 (3/3)	-	100.0 (1/1)		100.0 (4/4)	90.0 (9/10)	100.0 (4/4)	97.3 (36/37)	96.6 (57/59)
Adjacent type (*N*=34)	-	100.0 (1/1)	0 (0/3)		-	0 (0/2)	66.7 (6/9)	78.9 (15/19)	64.7 (22/34)
Without type (*N*=10)	-	-	55.6 (5/9)		-	-	100.0 (1/1)	-	60.0 (6/10)
Total	58.8 (10/17)				87.2 (75/86)				82.5 (85/103)

**4:** 影响EBUS-GS-TBLB对恶性病变诊断率的因素 Factors affecting the diagnosis rate of EBUS-GS-TBLB in malignant lesions

Variables		*N*	Diagnosed by EBUS-GS-TBLB [*n* (%)]	*χ*^2^	*P*
Lesion size				0.083	0.773
	≤20 mm	37	30 (81.1)		
	> 20 mm	66	55 (83.3)		
Probe location				20.372	0.000
	Within	59	57 (96.6)		
	Adjacent	34	22 (64.7)		
	Without	10	6 (60.0)		
Lesion location				1.377	0.848
	LUL	33	29 (87.9)		
	LLL	6	5 (83.3)		
	RUL	34	27 (79.4)		
	RML	12	9 (75.0)		
	RLL	18	15 (83.3)		
Bronchus sign				3.124	0.077
	Presence	34	32 (91.2)		
	Absence	69	53 (76.8)		
Lung field location				10.810	0.001
	Inner and middle band	73	66 (90.4)		
	Outer band	30	19 (63.3)		
Learning curve				0.000	0.997
	First 20 cases	20	16 (80.0)		
	After the 20 cases	83	69 (83.1)		
LUL: Left upper lobe; LLL: Left lower lobe; RUL: right upper lobe; RML: right middle lobe; RLL: right lower lobe.					

### 2.5 EBUS-GS-TBLB并发症

有1例（0.8%）患者刷检后出血量约100 mL，经局部支气管内注入冰盐水加肾上腺素，并给予血凝酶肌肉注射后血止，但未能完成后续活检及肺泡灌洗操作；23例（19.0%）患者术中出现一过性低氧血症，提高鼻导管氧流量后缓解；71例（58.7%）患者术后24 h内咳痰可见少量血丝，无需特殊处理；所有患者无气胸、感染等情况发生。

## 3 讨论

胸部CT可以比胸部X线检查发现更多的PPLs，其中相当一部分为良性病变^[9]^，明确病理诊断是治疗的基础。目前对于PPLs的活检方式包括：CT引导下TTNA以及经支气管镜活检技术（X线引导下TBLB、超细支气管镜活检术、EBUS-GS、电磁导航支气管镜等）。CT引导下TTNA对PPLs的诊断率为59%-96%，但并发症是不容忽视的问题，文献^[7]^报道术后气胸的发生率为15%-42%，其中4%-18%需要胸管引流，术后咯血的发生率为3%-12%。X线引导下TBLB操作简单，但其诊断率仅为30%-50%，难以满足临床需求。外径为2.8 mm的超细支气管镜比普通支气管镜能够更加深入远端支气管，在临床中常与其他VBN技术联合用于PPLs的诊断。电磁导航支气管镜对PPLs的诊断率为33%-96.8%，是ACCP推荐的PPLs诊断工具之一，但其价格昂贵，一定程度上限制了临床使用。1992年Hurter等^[10]^首先报道了支气管镜联合环扫支气管内超声（radial EBUS, r-EBUS）技术。2004年Kurimoto等^[6]^引入引导鞘技术，指出EBUS-GS能够提高PPLs的诊断率。r-EBUS被ACCP推荐用于PPLs的诊断^[7]^。

由于EBUS-GS仅能确认病灶，无法引导定位，临床中即使联合X线监视仍有11%-21%的病例因无法到达病灶而导致EBUS-GS失败。目前有两项针对VBN能否提高PPLs诊断率的前瞻性随机对照研究，Ishida等^[11]^认为EBUS-GS联合VBN不但能够提高诊断率（80.4% *vs* 67.0%），而且能够缩短操作时间（24 min *vs* 26.2 min）；而Asano等^[12]^的研究却显示，VBN未能提高PPLs的总诊断率（67.1% *vs* 59.9%），但在亚组分析中，VBN能够提高某些病灶（位于右肺上叶或肺野外带、X线下不可见）的诊断率。在回顾性研究中，EBUS-GS联合VBN技术对PPLs的诊断率为63.3%-84.4%，对于≤2 cm病灶的诊断率为44.4%-75.9%^[8]^。本研究中我们仅采用VBN进行导航，而未联合X线监视，对恶性病变的总诊断率及≤2 cm病灶的诊断率与上述研究结果相近。

在影响PPLs诊断率的因素中，当超声探头位于病灶内部或病灶直径 > 2 cm时EBUS-GS诊断率较高，这是目前多数研究^[13-16]^的结论。本研究中，超声探头位于病灶内部者诊断率高于位于病灶边缘或病灶外部者，与上述结论一致；虽然本组病灶直径 > 2 cm者诊断率高于直径≤2 cm者，但差异不具有统计学意义，这与Boonsarngsuk等^[17]^及Ishida等^[11]^的结果类似，考虑与本组病例总体数量偏少，特别是≤2 cm者比例偏低（33.7%）所造成的数据偏倚有关。

病灶位置与EBUS-GS诊断率相关。本研究中，位于肺野外带病灶的诊断率低于肺野内带及中带者，与Chavez等^[18]^及Tay等^[19]^的研究结果类似，原因考虑与邻近胸膜处末梢支气管更为纤细且鞘管及探头难以探入有关。鉴于CT引导下TTNA对邻近胸膜的病变具有较高的诊断率高和较低的并发症率^[7]^，建议此类病变可首先考虑TTNA。虽然本研究中不同肺叶病灶间诊断率无显著差异，但下叶病灶的诊断率低于上叶，这与Asano等^[12]^的研究类似，考虑与上叶较少受呼吸运动及心脏搏动影响有关，且采用外径4 mm的支气管镜，消除了普通支气管镜不易深入上叶段甚至亚段支气管的因素。Ali等^[20]^的荟萃分析显示，CT中存在支气管征（bronchus sign）的恶性病变，其EBUS-GS-TBLB的诊断率更高，这与本组病例的结果一致，考虑当出现支气管征时，鞘管更容易超选至目标病灶，从而更容易获得病理标本。

Boonsarngsuk等^[17]^的研究指出，活检、刷检及肺泡灌洗中诊断率最高的方法是活检，若将三者联合则能进一步提高诊断率。而在本研究中，尽管三种方法联合的诊断率最高，但就单一方式而言，刷检却是诊断率最高的方法。且需要注意的是本组恶性病例中组织学标本获取率偏低（70%），低于CT引导下TTNA的组织学标本获取率^[7]^，与其他采用EBUS-GS技术的获取率相差不大^[8, 11, 12]^。考虑与本研究未能联合使用X线监视，无法判断活检钳张合程度，导致取样标本较小病理诊断困难，甚至有时无法获得标本有关。虽然联合细胞学标本能够使得大部分病例获得恶性诊断，但目前肺癌治疗向着精准化及个体化发展，组织学标本能够为下一步药物治疗提供大量的信息，因此如何提高组织标本获取率，包括开发无需X线配合的新型活检钳也是今后该技术的改进之处。此外，临床实践中对于合并纵隔或肺门淋巴结肿大的病例，可考虑联合实施淋巴结活检，不但有可能进一步提高诊断比例，还能够同时完成肿瘤性病变的临床分期。

EBUS-GS对于良性病变的诊断率低于恶性病变，文献^[21]^报道其准确率为29%-69%，但各研究对于良性病变的定义不尽相同，本研究将EBUS-GS病理明确提示良性病变（如结核、结节病等）者归为EBUS-GS确诊病例，而将病理提示非特异性炎症、纤维化等归为未能确诊病例。我们建议在临床实践中亦应当按照这一原则对待EBUS-GS结果，必要时联合其他检查手段，避免误诊漏诊发生。

EBUS-GS并发症极低，文献报道其总并发症发生率约为1.5%，其中气胸最为常见，发生率为0-7.5%，其中仅有0.6%的患者需要胸管引流，相关感染的发生率约为0.5%，而大量出血或操作相关死亡病例未见报道^[13, 16, 22]^。本研究中，仅有1例出血偏多，但经内镜下处理顺利止血，未见气胸等并发症发生。

关于EBUS-GS-TBLB技术的培训，Eom等^[23]^的研究认为该技术较容易掌握，即便是对于初学者而言，仍有较高的诊断率。而Huang等^[24]^却认为，尽管该技术易于学习，但如果想保持较高的诊断水平，则需要至少3年或400例病例的学习经历。在本研究的恶性病例中，前20例的诊断率并未明显低于后续病例的诊断率，考虑与我们借助VBN技术、简化了初学者需要经验积累的过程有关。

本研究的不足之处在于，病例在最初接受EBUS-GS-TBLB操作时难免更倾向于选择那些预期操作成功率较高的病例，而做为回顾性研究，这种初始状态造成选择偏倚难以避免，因此未与同期接受CT引导下TTNA的病例进行比较。在后续研究中，随着对于该技术掌握熟练度的提高，拟针对符合条件的本院所有病例开展前瞻性随机对照研究，以期届时能够获得更加高质量的结果。另外，作为肿瘤专科医院，病种构成较为单一，本组病例中尚缺乏更多包括结核、结节病、特殊感染等良性病例，故对于EBUS-GS-TBLB在这类良性病变中诊断价值的探讨不足，有待后续病例的积累。

总之，EBUS-GS在PPLs诊断中具有诊断率高、并发症少的特点，即使在单纯使用VBN而不联合X线监视的条件下，其仍然是一种安全且有效的方法，有望在不具备X线监视条件的医疗单位得到推广。
